# Zika virus exposure affects neuron-glia communication in the hippocampal slices of adult rats

**DOI:** 10.1038/s41598-020-78735-y

**Published:** 2020-12-10

**Authors:** Larissa Daniele Bobermin, André Quincozes-Santos, Camila Leite Santos, Ana Paula M. Varela, Thais F. Teixeira, Krista Minéia Wartchow, Lílian Juliana Lissner, Amanda da Silva, Natalie K. Thomaz, Lucélia Santi, Walter O. Beys-da-Silva, Paulo M. Roehe, Patrícia Sesterheim, Jorge A. Guimarães, Carlos-Alberto Gonçalves, Diogo Onofre Souza

**Affiliations:** 1 Programa de Pós-Graduação em Ciências Biológicas: Bioquímica, Instituto de Ciências Básicas da Saúde, Universidade Federal do Rio Grande do Sul, Rua Ramiro Barcelos, 2600 – Anexo, Bairro Santa Cecília, Porto Alegre, RS 90035-003 Brazil; 2 Departamento de Bioquímica, Instituto de Ciências Básicas da Saúde, Universidade Federal do Rio Grande do Sul, Porto Alegre, RS Brazil; 3 Departamento de Microbiologia, Imunologia e Parasitologia, Instituto de Ciências Básicas da Saúde, Universidade Federal do Rio Grande do Sul, Porto Alegre, RS Brazil; 4 Faculdade de Farmácia, Universidade Federal do Rio Grande do Sul, Porto Alegre, RS Brazil; 5 Centro de Pesquisa Experimental, Hospital de Clínicas de Porto Alegre, Porto Alegre, RS Brazil; 6 Programa de Pós-Graduação em Biologia Celular e Molecular, Centro de Biotecnologia, Universidade Federal do Rio Grande do Sul, Porto Alegre, RS Brazil; 7 Centro de Cardiologia Experimental, Instituto de Cardiologia/Fundação Universitária de Cardiologia, Porto Alegre, RS Brazil

**Keywords:** Cytokines, Neurochemistry, Virology, Diseases of the nervous system, Glial biology, Neuroimmunology, Synaptic plasticity, Biochemistry, Diseases, Infectious diseases, Public health

## Abstract

Zika virus (ZIKV) infection during pregnancy was associated with microcephaly in neonates, but clinical and experimental evidence indicate that ZIKV also causes neurological complications in adults. However, the changes in neuron-glial communication, which is essential for brain homeostasis, are still unknown. Here, we report that hippocampal slices from adult rats exposed acutely to ZIKV showed significant cellular alterations regarding to redox homeostasis, inflammatory process, neurotrophic functions and molecular signalling pathways associated with neurons and glial cells. Our findings support the hypothesis that ZIKV is highly neurotropic and its infection readily induces an inflammatory response, characterized by an increased expression and/or release of pro-inflammatory cytokines. We also observed changes in neural parameters, such as adenosine receptor A2a expression, as well as in the release of brain-derived neurotrophic factor and neuron-specific enolase, indicating plasticity synaptic impairment/neuronal damage. In addition, ZIKV induced a glial commitment, with alterations in specific and functional parameters such as aquaporin 4 expression, S100B secretion and glutathione synthesis. ZIKV also induced p21 senescence-associated gene expression, indicating that ZIKV may induce early senescence. Taken together, our results indicate that ZIKV-induced neuroinflammation, involving nuclear factor erythroid 2-related factor 2 (Nrf2) and nuclear factor κB (NFκB) pathways, affects important aspects of neuron-glia communication. Therefore, although ZIKV infection is transient, long-term consequences might be associated with neurological and/or neurodegenerative diseases.

## Introduction

Zika virus (ZIKV) is a single-stranded RNA flavivirus, originally identified in Uganda in 1947, which has currently received attention because may cause severe damage in the fetal brain following maternal infection during gestation. In Brazil, the 2015 ZIKV outbreak was associated with microcephaly in neonates exposed to the virus during pregnancy^1^. ZIKV markedly affects the cytoarchitecture of the brain, as well as induces neurochemical and molecular dysfunctions in neural cells^2–6^. Current clinical and experimental data indicate that ZIKV also affects the mature brain, causing neurological complications including Guillain-Barré and other myelitis and encephalitis-like illnesses^7–11^. However, the underlying neurochemical changes are still poorly understood. The hippocampus is a crucial brain structure for learning and memory, and hippocampal damage leads to the progression of severe psychological and cognitive impairments. Hippocampal neurogenesis, which persists throughout life, is affected by immune activity triggered by pathogens including ZIKV^3,12^.

Although neurons are the main focus of neuroscience research, glial cells are essential for the central nervous system (CNS), particularly because alterations in their functions can promote neuronal survival or death^13^. Astrocytes are able to sense and respond to changes in their surrounding microenvironment^14,15^. These cells also contribute to the formation and integrity of blood–brain barrier (BBB), affect axonal outgrowth and support neuronal metabolism and synaptic activity^16,17^. Moreover, astrocytes actively participate in neuron-glial communication, releasing trophic factors and inflammatory mediators^18,19^. With regard to ZIKV infection, astrocytes are potentially the first cells targeted by ZIKV in the CNS, since they present the AXL receptor, a candidate receptor to host ZIKV in the brain^20,21^. Indeed, microglial cells are the tissue-resident macrophage-like immune cells that continuously maintain an immune surveillance of the CNS^22^, thus they can mediate inflammatory response after ZIKV exposure. The production of pro-inflammatory cytokines and other signalling molecules by microglia and/or astrocytes is part of the defence system against invading pathogens in the brain and are particularly orchestrated by nuclear factor κB (NFκB) pathway^23,24^. Despite of both astrocytes and microglia presenting protective/defensive roles, these cells can shift their phenotype and release a wide range of harmful signalling molecules. As glial cells regulate tissue recovery after injury, dysfunctions in these cells may be associated with neurological outcomes of ZIKV and represent a risk factor for neurodegenerative diseases and neuropsychiatric disorders^13,25,26^.

In line with this, the aim of this study was to evaluate the changes in neuronal and glial functions in an ex vivo experimental model of acute hippocampal slices from adult rats submitted to ZIKV infection. Thus, we determined neurochemical parameters, inflammatory response, cellular and molecular pathways, including nuclear factor erythroid 2-related factor 2 (Nfr2) and NFκB, which are strongly associated with neuron-glia communication. This interaction is a crucial process for CNS homeostasis, and our findings provide the first evidence that ZIKV alters neuron-glia communication.

## Results

### ZIKV infected hippocampal slices from adult rats

**Figure 1:**
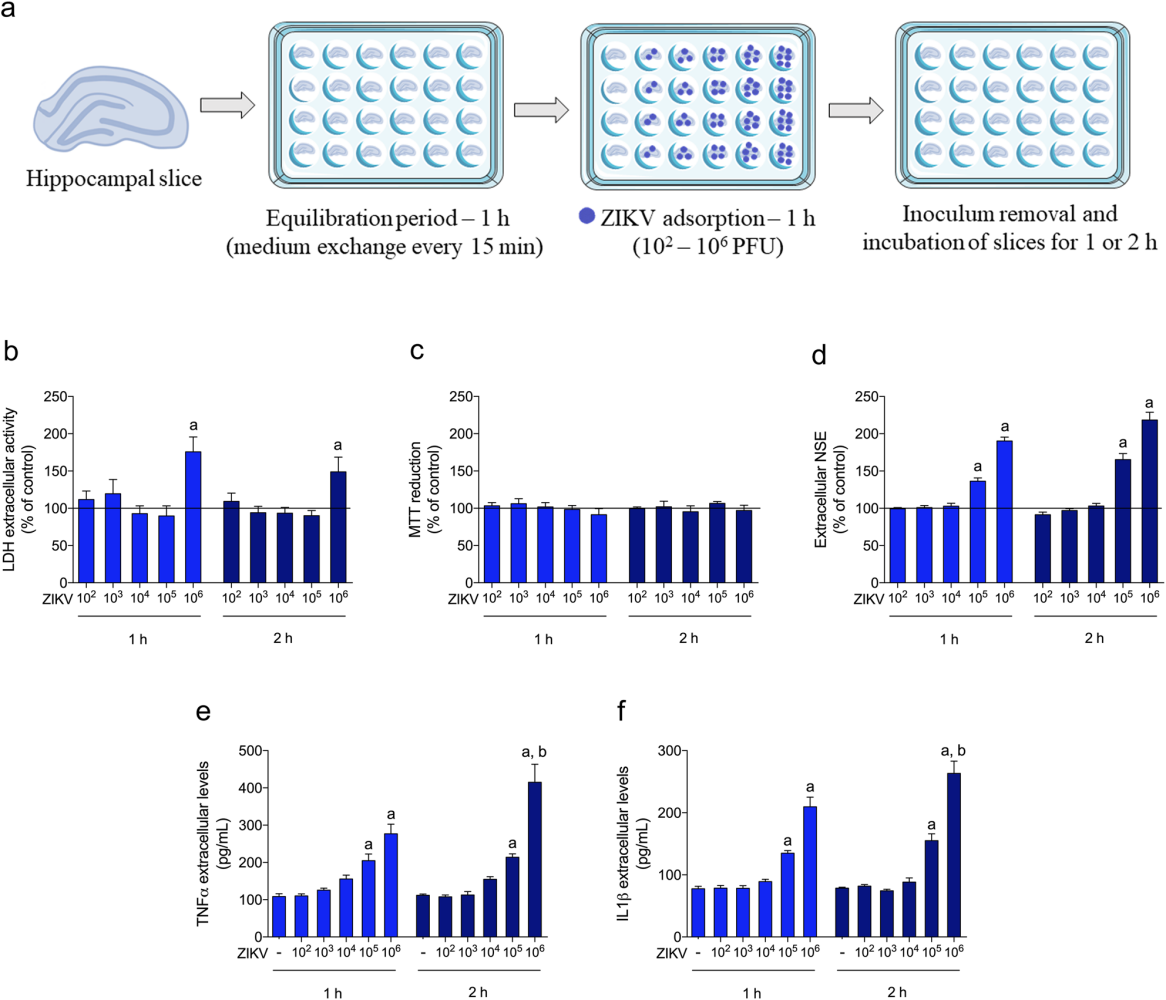
Effects of ZIKV on the hippocampus of adult rats. (**a**) Experimental design of ZIKV infection. Hippocampal slices (0.3 mm thickness) were obtained from adult rats and were maintained for an equilibration period of up to 1 h (cellular recovery), following ZIKV adsorption (10^2^–10^6^ PFU) or non-infection control for 1 h. The inoculum was then washed out, and the slices were maintained for an additional 1 or 2 h; (**b**) extracellular LDH activity; (**c**) MTT reduction; (**d**) extracellular NSE activity. The line indicates the non-infection control value, assumed as 100%. Data represent means ± SEM of at least four experimental determinations performed in quadruplicate, analyzed by one-way ANOVA followed by Tukey’s test. Values of P < 0.05 were considered significant (*a* indicates difference from control condition; *b* indicates difference between 1 and 2 h incubations after ZIKV inoculation). The release of TNFα (**e**) and IL1β (**f**) was evaluated using the extracellular medium of hippocampal slices after incubation with ZIKV (10^2^–10^6^ PFU) or non-infection control (first column in the graphs). Data represent means ± SEM of at least four experimental determinations performed in quadruplicate, analyzed by one-way ANOVA followed by Tukey’s test. Values of P < 0.05 were considered significant (*a* indicates difference from control condition; *b* indicates difference between 1 and 2 h incubations after ZIKV inoculation).

**Figure 2:**
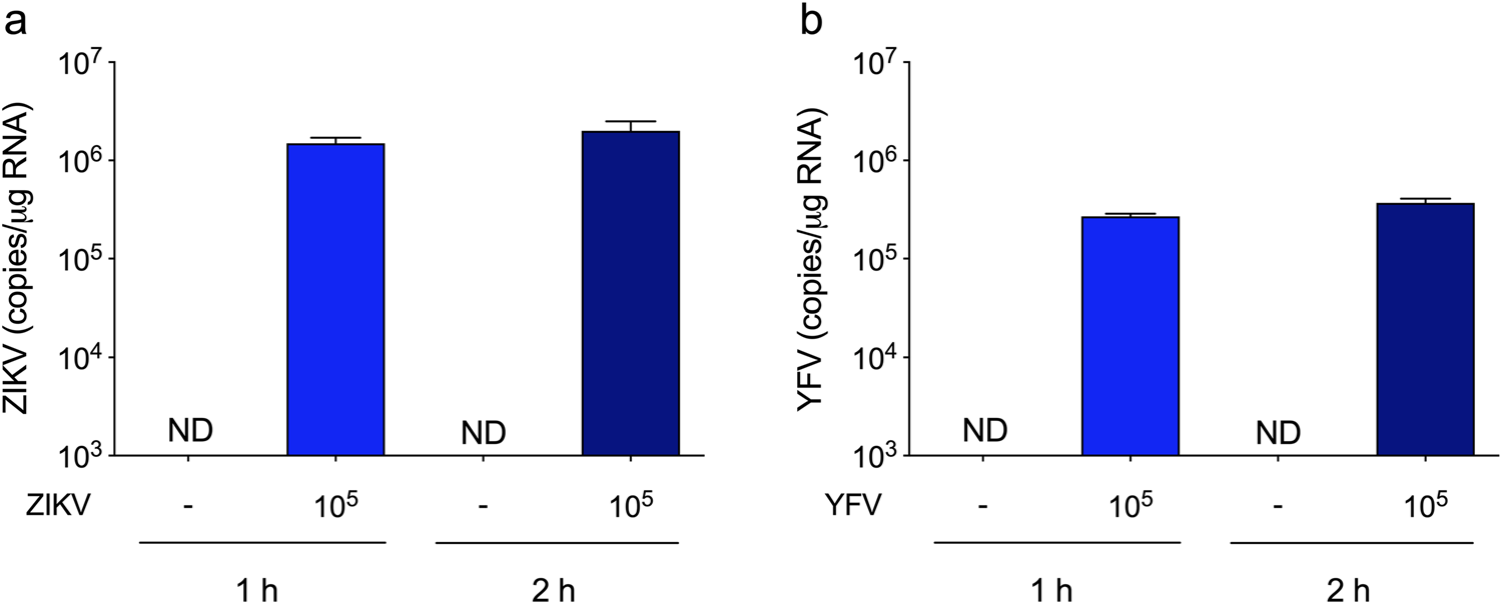
Viral copies detection in hippocampal slices. Hippocampal slices were infected with 10^5^ PFU of ZIKV or YFV (used as a comparative Flavivirus) according the experimental design depicted in Fig. 1a. Non-infection controls were simultaneously carried out. Quantitative PCR for ZIKV (**a**) and YFV (**b**) was performed to determinate viral copies in the hippocampal slice after 1 h or 2 h incubation. Bars represent means ± SEM of three experimental determinations (*ND* not detectable).

We first investigated whether ZIKV17 could affect cellular viability and integrity of hippocampal slices. Thus, hippocampal slices were incubated for 1 or 2 h with 10^2^ to 10^6^ plaque-forming units (PFU) as shown in the Fig. 1a, and it was observed loss in cellular integrity, measured by extracellular lactate dehydrogenase (LDH), at 10^6^ PFU (Fig. 1b), without any change in MTT reduction assay (Fig. 1c). In addition, a significant increase in extracellular neuron-specific enolase (NSE) (Fig. 1d, Table 2) was observed, indicating neuron death at both exposure times. The viral doses of 10^5^ and 10^6^ PFU induced tumour necrosis factor α (TNFα) and interleukin (IL) 1β release (Fig. 1e,f, Table 1), but extracellular NSE increased without change in LDH only at 10^5^ PFU. Therefore, we performed the subsequent experiments using 10^5^ PFU. Then, we evaluated the presence of ZIKV copies in acute hippocampal slices. Notably, we incubated hippocampal slices with 10^5^ PFU and after 1 or 2 h of the inoculum removal (as represented in the Fig. 1a), the ZIKV copies were maintained with a slight exponential increment (from 10^5^ to 10^6^) in the hippocampal slices (Fig. 2a), reinforcing its ability to infect neural cells. We used yellow fever virus (YFV17DD) as a comparative Flavivirus, and after incubating hippocampal slices with 10^5^ PFU, the viral copies of YFV after the same times of incubations did not increase as much as the ZIKV (Fig. 2b).

**Table 1:** Effects of ZIKV on inflammatory/redox signaling and associated pathways.

Parameter	Control 1 h (A)	ZIKV 1 h (B)	Control 2 h (C)	ZIKV 2 h (D)	Method	F	P	Post hoc
**Inflammatory signaling**								
TNFα (pg/mL)	108 ± 8	205 ± 18	111 ± 4	214 ± 9	ELISA	27.2	< 0.0001	B ≠ A; D ≠ C
TNFα (mRNA)	1.0 ± 0.09	2.1 ± 0.12	0.9 ± 0.08	2.7 ± 0.15	RT-PCR	60.7	< 0.0001	B ≠ A; D ≠ C; D ≠ B
TNFR1 (mRNA)	1.0 ± 0.06	1.0 ± 0.04	1.0 ± 0.04	1.1 ± 0.10	RT-PCR	0.374	> 0.05	
IL1β (pg/mL)	77 ± 4	135 ± 4	78 ± 2	155 ± 11	ELISA	50.66	< 0.0001	B ≠ A; D ≠ C
IL1β (mRNA)	1.0 ± 0.04	2.1 ± 0.10	1.0 ± 0.07	3.2 ± 0.22	RT-PCR	64.71	< 0.0001	B ≠ A; D ≠ C; D ≠ B
IL1R1 (mRNA)	1.0 ± 0.09	1.0 ± 0.12	1.1 ± 0.12	1.2 ± 0.13	RT-PCR	0.617	> 0.05	
IL6 (pg/mL)	70 ± 3	74 ± 3	69 ± 2	98 ± 2	ELISA	27.19	< 0.0001	D ≠ C; D ≠ B
IL10 (pg/mL)	23 ± 1	23 ± 1	22 ± 1	7 ± 1	ELISA	43.13	< 0.0001	D ≠ C; D ≠ B
MCP1 (pg/mL)	27 ± 1	25 ± 2	26 ± 1	44 ± 4	ELISA	16.58	< 0.0001	D ≠ C; D ≠ B
HMGB1 (mRNA)	1.0 ± 0.03	0.9 ± 0.08	0.9 ± 0.06	1.1 ± 0.09	RT-PCR	1.252	> 0.05	
COX2 (mRNA)	1.0 ± 0.04	1.7 ± 0.06	0.9 ± 0.06	2.5 ± 0.13	RT-PCR	81.97	< 0.0001	B ≠ A; D ≠ C; D ≠ B
TLR2 (mRNA)	1.0 ± 0.06	1.2 ± 0.14	1.0 ± 0.08	2.2 ± 0.17	RT-PCR	20.46	< 0.0001	D ≠ C; D ≠ B
TLR4 (mRNA)	1.0 ± 0.07	1.0 ± 0.10	1.0 ± 0.06	1.2 ± 0.10	RT-PCR	1.146	> 0.05	
NFκB p65 (mRNA)	1.0 ± 0.05	1.7 ± 0.13	0.9 ± 0.05	2.3 ± 0.21	RT-PCR	25.77	< 0.0001	B ≠ A; D ≠ C; D ≠ B
NFκB p50 (mRNA)	1.0 ± 0.07	0.9 ± 0.07	1.1 ± 0.04	1.4 ± 0.10	RT-PCR	8.974	0.0006	D ≠ C; D ≠ B
**Redox signaling**								
Nrf2 (mRNA)	1.0 ± 0.02	0.7 ± 0.04	1.1 ± 0.03	0.5 ± 0.04	RT-PCR	69.18	< 0.0001	B ≠ A; D ≠ C; D ≠ B
HO1 (mRNA)	1.0 ± 0.03	0.7 ± 0.03	1.0 ± 0.03	0.4 ± 0.02	RT-PCR	119.1	< 0.0001	B ≠ A; D ≠ C; D ≠ B
iNOS (mRNA)	1.0 ± 0.07	1.0 ± 0.10	0.9 ± 0.06	1.7 ± 0.10	RT-PCR	19.16	< 0.0001	D ≠ C; D ≠ B
SOD1 (mRNA)	1.0 ± 0.07	0.6 ± 0.06	1.0 ± 0.02	0.5 ± 0.08	RT-PCR	20.95	< 0.0001	B ≠ A; D ≠ C
SOD2 (mRNA)	1.0 ± 0.02	0.6 ± 0.02	1.1 ± 0.03	0.4 ± 0.03	RT-PCR	129.3	< 0.0001	B ≠ A; D ≠ C; D ≠ B
GSH (%)	100 ± 12	100 ± 23	100 ± 3	158 ± 20	FA	3.463	0.026	D ≠ C
GCL (mRNA)	1.0 ± 0.06	1.1 ± 0.07	1.1 ± 0.05	2.0 ± 0.09	RT-PCR	42.11	< 0.0001	D ≠ C; D ≠ B
**Other pathways**								
PI3K (mRNA)	1.0 ± 0.04	0.9 ± 0.06	0.9 ± 0.04	0.5 ± 0.05	RT-PCR	25.71	< 0.0001	D ≠ C; D ≠ B
PI3K (protein)	100 ± 33	76 ± 29	45 ± 10	61 ± 23	WB	0.789	> 0.05	
Akt (mRNA)	1.0 ± 0.08	1.0 ± 0.05	1.1 ± 0.05	1.0 ± 0.08	RT-PCR	0.325	> 0.05	
Akt (protein)	100 ± 19	92 ± 19	108 ± 20	103 ± 13	WB	0.140	> 0.05	
p21 (mRNA)	1.0 ± 0.04	0.9 ± 0.05	1.0 ± 0.03	1.5 ± 0.04	RT-PCR	39.51	< 0.0001	D ≠ C; D ≠ B
SIRT1 (mRNA)	1.0 ± 0.08	0.8 ± 0.09	0.9 ± 0.08	0.9 ± 0.10	RT-PCR	0.765	> 0.05	

Hippocampal slices from adult Wistar rats were incubated with medium containing ZIKV (10^2^ to 10^6^ PFU) for an adsorption period of 1 h. Afterwards, this medium was exchanged for fresh saline medium for 1 h or 2 h, and the parameters presented in Table were measured, as described in the “Methods”: section. Data are expressed as: (i) pg/mL for ELISA assays; (ii) fold increase for mRNA levels (RT-PCR); (iii) percentages of control for protein levels (Western blotting—WB); (iv) GSH content (fluorimetric assay—FA). Differences among groups were statistically analyzed using one-way analysis of variance (ANOVA), followed by Tukey’s test (n = 6 per group, except for the WB analysis in which at least three experimental determinations were performed). Values of P < 0.05 were considered significant. P values are indicated in the Table. Treatment groups that differ significantly are listed in the Post hoc column. The representative images of WB are in the Supplementary Material (Fig. S1).

### Signalling mechanisms underlying ZIKV-induced inflammatory response and redox imbalance

In order to characterize the inflammatory response, we measured mRNA expression levels of TNFα and IL1β, which increased after 1 and 2 h of ZIKV exposure (Table 1). However, their receptors, TNFR1 and IL1R1, respectively, did not change. In addition, the pro-inflammatory markers, IL6 and monocyte chemoattractant protein 1 (MCP1), increased at 2 h of ZIKV exposure, while the anti-inflammatory cytokine IL10 significantly decreased. ZIKV did not affect the mRNA expression of an alarmin that is specific to immune cells, high mobility group box 1 (HMGB1) (Table 1), but significantly decreased the secretion of the S100B, assumed to be an astrocyte-derived alarmin^27^ (Table 2).

**Table 2:** Effects of ZIKV on neurotrophic factors, adenosine receptors, and specific neuron and glial parameters.

Parameter	Control 1 h (A)	ZIKV 1 h (B)	Control 2 h (C)	ZIKV 2 h (D)	Method	F	P	Post hoc
**Neurotrophic signaling**								
BDNF (pg/mL)	64 ± 2	87 ± 4	62 ± 2	39 ± 4	ELISA	42.82	< 0.0001	B ≠ A; D ≠ C; D ≠ B
GDNF (pg/mL)	71 ± 4	95 ± 5	68 ± 5	115 ± 2	ELISA	28.57	< 0.0001	B ≠ A; D ≠ C; D ≠ B
VEGF (mRNA)	1.0 ± 0.06	1.7 ± 0.12	1.0 ± 0.07	2.2 ± 0.10	RT-PCR	40.19	< 0.0001	B ≠ A; D ≠ C; D ≠ B
**Adenosine receptors**								
A1 (mRNA)	1.0 ± 0.06	1.1 ± 0.06	0.9 ± 0.05	0.9 ± 0.04	RT-PCR	3.077	> 0.05	
A2a (mRNA)	1.0 ± 0.02	1.1 ± 0.10	0.9 ± 0.05	1.6 ± 0.05	RT-PCR	28.03	< 0.0001	D ≠ C; D ≠ B
A2b (mRNA)	1.0 ± 0.04	1.0 ± 0.03	1.0 ± 0.03	1.0 ± 0.02	RT-PCR	0.120	> 0.05	
A3 (mRNA)	1.0 ± 0.05	1.1 ± 0.05	1.1 ± 0.04	1.1 ± 0.06	RT-PCR	1.122	> 0.05	
**Specific glial parameters**								
GFAP (mRNA)	1.0 ± 0.05	0.8 ± 0.05	0.9 ± 0.07	0.9 ± 0.07	RT-PCR	0.656	> 0.05	
GFAP (protein)	100 ± 7	103 ± 8	87 ± 21	75 ± 29	WB	0.551	> 0.05	
Vimentin (mRNA)	1.0 ± 0.18	0.7 ± 0.03	0.8 ± 0.09	1.5 ± 0.19	RT-PCR	7.798	0.0006	D ≠ C; D ≠ B
Nestin (mRNA)	1.0 ± 0.11	0.8 ± 0.05	0.7 ± 0.03	1.4 ± 0.10	RT-PCR	9.250	0.0005	D ≠ C; D ≠ B
AQP4 (mRNA)	1.0 ± 0.06	1.1 ± 0.05	1.0 ± 0.10	1.7 ± 0.07	RT-PCR	19.58	< 0.0001	D ≠ C; D ≠ B
GLT1 (mRNA)	1.0 ± 0.08	1.1 ± 0.10	0.8 ± 0.07	0.9 ± 0.08	RT-PCR	1.98	> 0.05	
GLAST (mRNA)	1.0 ± 0.05	1.1 ± 0.08	0.8 ± 0.08	0.9 ± 0.10	RT-PCR	2.437	> 0.05	
GS (mRNA)	1.0 ± 0.10	1.1 ± 0.04	0.9 ± 0.06	1.0 ± 0.12	RT-PCR	0.388	> 0.05	
S100B (%)	100 ± 6	112 ± 13	100 ± 13	38 ± 4	ELISA	10.48	< 0.0001	D ≠ C; D ≠ B
**Specific neuron markers**								
NSE (%)	100 ± 2	136 ± 4	100 ± 2	165 ± 8	LA	67.25	< 0.0001	B ≠ A; D ≠ C
EAAC1 (mRNA)	1.0 ± 0.07	0.9 ± 0.04	1.0 ± 0.06	1.1 ± 0.10	RT-PCR	1.842	> 0.05	
NMDA-R1 (protein)	100 ± 23	100 ± 21	52 ± 22	57 ± 27	WB	1.258	> 0.05	
Synaptophysin (protein)	100 ± 11	79 ± 12	101 ± 7	112 ± 22	WB	1.027	> 0.05	
β-tubulin III (protein)	100 ± 11	129 ± 23	106 ± 20	93 ± 18	WB	0.754	> 0.05	

Hippocampal slices from adult Wistar rats were incubated with medium containing ZIKV (10^2^ to 10^6^ PFU) for an adsorption period of 1 h. Subsequently, this medium was exchanged for fresh saline medium for 1 h or 2 h, and the parameters presented in Table were measured, as described in the “Methods” section. Data are expressed as: (i) pg/mL for ELISA assays, except for S100B ELISA, expressed as the percentage of control; (ii) fold increase for mRNA levels (RT-PCR); (iii) percentages of control for protein levels (Western blotting—WB). Differences among groups were statistically analyzed using one-way analysis of variance (ANOVA), followed by Tukey’s test (n = 6 per group, except for the WB analysis in which at least three experimental determinations were performed). Values of P < 0.05 were considered significant. P values are indicated in the Table. Treatment groups that differ significantly are listed in the Post hoc column. The representative images of WB are in the Supplementary Material (Fig. S1).

Other markers associated with inflammatory signalling were evaluated, including mRNA encoding NFκB p65, NFκB p50 and cyclooxygenase (COX) 2, which increased with ZIKV exposure (Table 1). As such, NFκB may be a key element in controlling ZIKV-induced damage in hippocampal slices, where it regulates the expression of cytokines, chemokines and immunoreceptors^28^. To elucidate the response triggered by ZIKV, we measured levels of mRNA encoding for toll-like receptors (TLR) 2 and 4, which can bind virus, bacteria, pro-inflammatory cytokines and alarmins, such as HMGB1^12^. However, only TLR2 expression was increased by ZIKV (Table 1).

ZIKV exposure increased GSH levels and its rate-limiting enzyme, glutamate-cysteine ligase (GCL) (Table 1). This increase may be associated to astroglial reactivity, as GSH is the major brain antioxidant compound, whose production depends on astrocyte activity^29^. Conversely, mRNA expression levels of the enzymes superoxide dismutase (SOD) 1 and 2 decreased, while inducible nitric oxide synthase (iNOS) increased. In addition, the expression of Nrf2 decreased in a time-dependent manner, as well as the mRNA levels of heme oxygenase 1 (HO1), a fundamental defence mechanism for cells exposed to challenge stressors (Table 1).

Besides these pathways, Table 1 also displays that mRNA levels of phosphoinositide-3-kinase (PI3K) decreased, but its downstream signal, Akt, did not change, as well as phospho-PI3K immunocontent. In addition, p21 senescence-associated gene expression increased, indicating that ZIKV may induce early senescence^30^. However, sirtuin 1 (SIRT1), a pathway that may counteract the inflammatory response and oxidative redox imbalance, did not change.

### ZIKV modulated neurotrophic factor release and adenosine receptor expression

Neurotrophic factors have been shown to modulate synaptic plasticity, neural response and recovery. ZIKV induced a fast increase in brain-derived neurotrophic factor (BDNF) and glial cell-derived neurotrophic factor (GDNF) release, as well as vascular endothelial growth factor (VEGF) mRNA levels (Table 2). However, 2 h afterwards, we observed only a decrease in BDNF secretion.

Neurotrophic factors/synaptic plasticity are closely associated with brain adenosine receptors, which are also involved in neuroinflammation^31^. In our experimental model, we observed that ZIKV increased only the expression of adenosine receptor A2a (Table 2).

### ZIKV changed specific neuron/glial parameters

To characterize the different commitment between neurons and glial cells, we examined some specific parameters. With regard to glial markers, ZIKV quickly increased the mRNA expression of aquaporin 4 (AQP4) (Table 2), whose role in neuroinflammation and neurodegenerative diseases has been increasingly highlighted^32^. As commented above, S100B secretion decreased after ZIKV exposure; however, the expression of glial fibrillary acidic protein (GFAP) and other specific markers such as glutamate transporters (GLAST and GLT1) and glutamine synthetase (GS) did not change in the acute presence of ZIKV (Table 2). In contrast, ZIKV exposure at 2 h increased the mRNA expression of vimentin and nestin, two other filament intermediary proteins found in astrocytes.

Regarding neurons, although there was an increase in extracellular NSE levels, there was no alteration in the expression of other specific markers, such as the EAAC1 glutamate transporter, N-methyl-D-aspartate receptor 1 (NMDA-R1), synaptophysin and β-tubulin III, after ZIKV exposure (Table 2).

## Discussion

**Figure 3:**
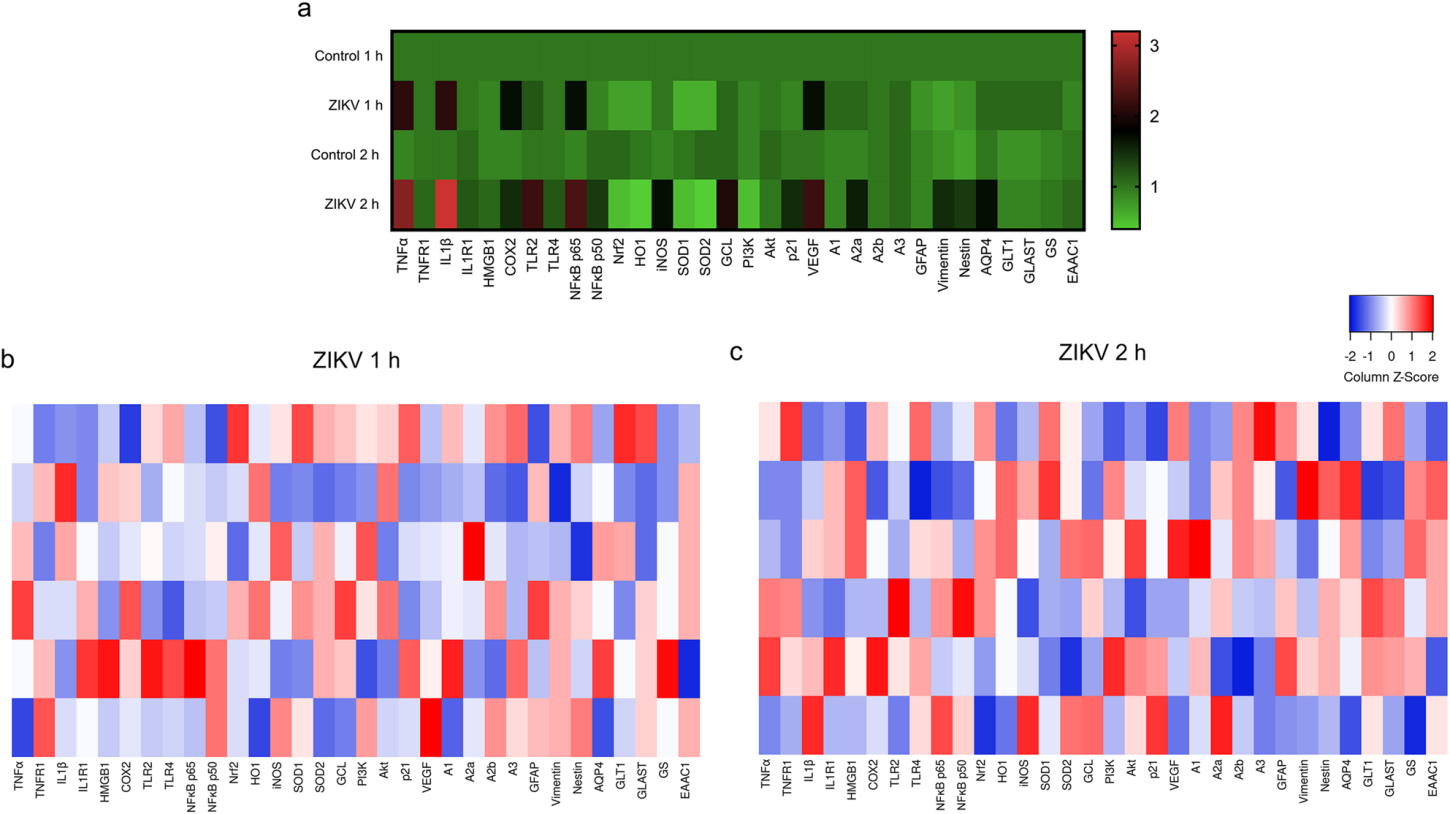
Heatmaps representing gene expression after acute ZIKV exposure in hippocampal slices. (**a**) The relative mRNA expression in the different experimental groups. Each square represents the mean of the group and the color scale is shown at the right. The gene expressions of hippocampal slices after 1 h (**b**) or 2 h (**c**) ZIKV exposure were analyzed as Z-score values. Each square represents a biological replicate (n = 6 per group).

Although ZIKV was initially associated with microcephaly in neonates and developmental anomalies, increasing evidence has shown that it can replicate in adult brain tissue, being able to affect synapses and induce cognitive deficits^3,7–10^. Herein, for the first time, we reported that ZIKV is able to quickly infect hippocampal slices from adult rats, acutely causing a wide range of cellular and molecular alterations, regarding to redox, inflammatory and neurotrophic parameters (Fig. 3). These alterations can affect neuron-glia communication, which is crucial to brain homeostasis. Thus, although ZIKV infection can be transient, it can induce significant changes in the adult brain functionality, whose long-term consequences are unknown, but might become an important health concern^25,26^.

Hippocampus is a crucial region involved in learning and long-term memory processes. This brain structure is susceptible to endogenous and/or exogenous factors that can lead synaptic plasticity impairment, which is often manifested in neurodegenerative diseases^33,34^. Interestingly, hippocampus seems to be an important target region for ZIKV. Previously, it has been demonstrated that adult neural stem cells from hippocampus are vulnerable to ZIKV, which causes cell death and reduced proliferation^35^. Although it has been suggested that glial cells, particularly astrocytes, are mostly affected by ZIKV because it can bind to AXL receptor^20,21^, recent results in cultured mouse hippocampal slices indicate acute ZIKV infection in neurons and not in astrocytes, contrary to expectations based on entry via AXL receptors^3^.

Acute ZIKV exposure in adult hippocampal slices caused a markedly inflammatory response, which can alter neuronal synaptic communication. Notably, neuroinflammation is a common point between congenital microcephaly in newborns and neurological complications in childhood and adults^36^. Although glial cells are the main cells responsible for producing and releasing inflammatory mediators, we do not know the origin of these cytokines at this time, and neurons could be also considered as a source. Accordingly, ZIKV-infected neurons in culture exhibit increased levels of TNFα and IL1β^37^. Moreover, as previously reported in viral encephalitis, neurons can be primary targets releasing mediators by informing neighbouring cells and attracting immune cells from the blood^38^. During ZIKV infection, the permeability of the BBB can increase as a consequence of the overproduction of cytokines, thus favouring the access of peripheral cells and ZIKV to the brain^36^. In line with this, peripheral blood mononuclear cells were identified as important cellular targets of American ZIKV strain infection, and for promoting ZIKV spread^39^. It is important to note that astrocytes are functional elements in the BBB, thus these cells can contribute to propagation and progress of ZIKV infection, causing injury of neural cells through direct infection-induced and/or indirect immune-mediated mechanisms^40–42^.

Astrocytes play an important role in the CNS antioxidant defence, since they can provide glutathione (GSH) and superoxide dismutase (SOD) to neurons^43,44^. Changes in this function can impair the adult brain, contributing to further neurological manifestations related to ZIKV infection. Interestingly, we observed an increase in both glutamate-cysteine ligase (GCL) expression and in the GSH content, probably as an early compensatory mechanism in response to ZIKV exposure. In contrast, ZIKV acutely modulated the expression of other genes related to redox homeostasis/oxidative stress; particularly, there was a downregulation of HO1, SOD1 and SOD2, and an upregulation of iNOS. Accordingly, a recent study in human iPSC-derived astrocytes showed that ZIKV infection induced oxidative stress, mitochondrial failure and DNA damage^2^. ZIKV-induced dysfunctions in mitochondrial activity are also potentially associated with excitotoxicity. Notably, ZIKV-infected neurons release increased levels of glutamate^37^. Although we did not observe changes in the expressions of astrocytic and neuronal glutamate transporters, their activity may be impaired by oxidation^45^, potentially causing excitotoxicity. In addition, this process is potentiated by Ca^2+^ release from the mitochondria and endoplasmic reticulum, and ZIKV can interfere with Ca^2+^ uptake by mitochondria^2^. However, at least acutely, ZIKV did not modulate NMDA-R1 protein levels.

The differential expression profile observed for TLRs may indicate differences in infection and immunity in response to ZIKV. Although TLRs trigger inflammatory and antiviral responses, they can also modulate adult hippocampal neurogenesis^12^. In the context of acute hippocampal injury, neurotrophic factors have been shown to modulate neural response and recovery^46^. The decrease of BDNF could contribute to impair synaptic plasticity. However, synaptophysin, a pre-synaptic protein widely used as a marker of synaptic plasticity, was not affected at this short time. In addition, considering that A2a receptor is associated with synaptic plasticity and inflammatory process^47,48^, the increased expression of this receptor induced by ZIKV, can be a possible link between ZIKV, neuroinflammation and long-term neurological diseases^26^. It is important to note that the decrease in BDNF and increase in A2a perhaps favour the release of glutamate in neurons^49^, a common event associated with excitotoxicity and age-related diseases. In this sense, ZIKV-induced an upregulation of the senescence marker p21 in hippocampal slices. Moreover, the protein S100B is frequently used as a marker of astrocyte activation, and can produce either neurotrophic or deleterious effects, depending on the concentration^50^, showed a decreased release, suggesting that ZIKV also affects trophic signalling mediated by astrocytes.

Since there is a close relationship between inflammation and redox signalling, we investigated classical pathways that interconnect these events in neural cells, namely NFκB and Nfr2. Nfr2 is a transcription factor involved in the adaptive response to cellular stress, including the oxidative stress induced during the inflammatory response, and their target genes, which induce antioxidant enzyme production, GSH synthesis and eventually inhibit cytokine-mediated inflammation^51^. Increasing evidence has suggested that activation of Nrf2 is more restricted to astrocytes^52^. While the increased expression and activity of Nrf2 are associated with protective mechanisms, deficiencies have been correlated with exacerbated astrogliosis, GFAP expression and worsening of inflammatory parameters in a mouse model of neurodegeneration, as well as with impaired neuronal differentiation of neural stem cells in the subgranular zone of the hippocampus^53^. Furthermore, in the CNS, cell-type specific pathological roles of NFκB have been described, including aberrant synapse to nuclear communication in neurons, and glial activation leading to chronic neuroinflammation, with consequent neuronal cell death^28^. Considering that there is an interplay between inflammatory and oxidative signals, in which Nrf2 depletion enhances NFκB signaling, and the latter eventually modulates Nrf2 transcription^51^, data indicate that the transcription factors, NFκB and Nrf2, may be important mechanistic partners in the altered neuron-glial communication observed after ZIKV exposure.

**Figure 4:**
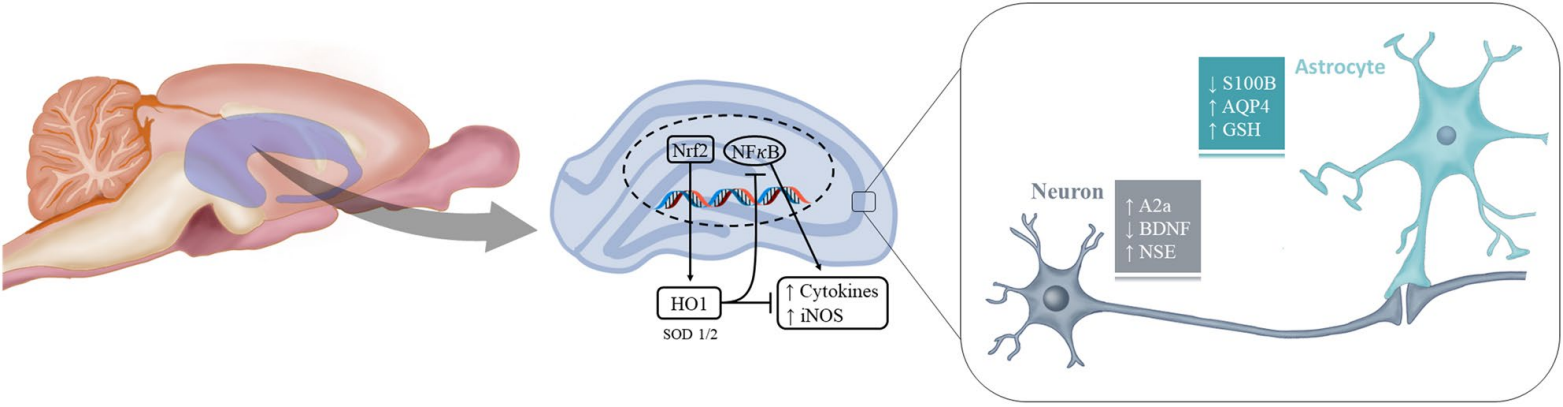
Schematic illustration of some cellular targets of ZIKV in neural cells. Our data reinforce the strong neurotropism of ZIKV, which was able to readily increase the expression and/or release of pro-inflammatory mediators, such as cytokines and iNOS. Inflammatory response is mainly coordinated by NFκB. In contrast, Nrf2 and its transcriptional products, such as HO1, are important regulators of adaptive responses to cellular stresses. HO1 is able to counteract inflammatory response and NFκB transcription activity. However, both Nrf2 and HO1 were downregulated by ZIKV exposure. More specific neuronal and astroglial ZIKV-induced effects could also be observed. A decrease in BDNF release, an increase in NSE and in A2a receptor gene expression can be mainly attributed to neurons (although A2a can be also expressed by astrocytes and microglia). Moreover, a decrease in S100B release, as well as an increase in mRNA levels of AQP4 and in GSH content can indicate an acute ZIKV-induced glial commitment in the hippocampus of adult rats.

Our data support the hypothesis that ZIKV is highly neurotropic and its infection readily increases the expression of intermediate filaments, vimentin and nestin, found in astrocytes and precursor neural cells, which could contribute to the aberrant brain cytoarchitecture found in fetuses exposed to ZIKV. Consistent with this increased expression of vimentin and nestin, evidence has suggested that an immature phenotype may re-emerge in astrocytes in the pathological adult brain, in an effort to promote synapse remodelling^54^. Moreover, and just as importantly, our study has generated data to indicate that ZIKV-induced neural damage occurs in the mature brain, particularly in the hippocampus. Based on GFAP expression and content, astrocytes may appear to be unaffected by acute ZIKV exposure. However, other specific and functional parameters such as AQP4, S100B secretion, GSH biosynthesis, and underlying glial signalling pathways indicate acute glial commitment (Fig. 4). In summary, our findings from ex vivo hippocampal slices acutely exposed to ZIKV, indicate that ZIKV-induced neuroinflammation affects important aspects of neuron-glia communication that are commonly affected in neurodegenerative diseases.

## Methods

### ZIKV

Brazilian ZIKV strain 17 (ZIKV17, 6 passages in VERO cells; kindly provided by Dr. E. Durigon, Institute of Biomedical Sciences, University of São Paulo^55^—GenBank sequence accession number MH882541.1) and YFV vaccinal strain 17DD (produced by Instituto de Tecnologia em Imunobiológicos Bio-Manguinhos), were propagated in African green monkey kidney E6 (VERO E6) cells (ATCC CRL-1586). Viruses were also titrated on VERO E6, using a standard plaque assay. Viral stocks were maintained at − 80 °C.

### Animals

Male Wistar rats (30 days old) were obtained from the breeding colony of the Department of Biochemistry (Federal University of Rio Grande do Sul, Porto Alegre, Brazil) and maintained under a controlled environment (12-h light/12-h dark cycle; 22 ± 1 °C; ad libitum access to food and water). All animal experiments were performed in accordance with the National Institutes of Health (NIH) Guide for the Care and Use of Laboratory Animals and were approved by the Animal Care and Use Committees of Federal University of Rio Grande do Sul (process numbers 24419 and 36949) and Instituto de Cardiologia/Fundação Universitária de Cardiologia (CEUA-IC/FUC 001/2018).

### Hippocampal slices preparation

The hippocampi were dissected from brain and transversally sliced (0.3 mm thickness) using a Mcllwain Tissue Chopper^56^. Slices were then transferred immediately into 24-well culture plates (one slice per well), with each well containing 0.3 mL of physiological saline medium (composition in mM: 120 NaCl; 2 KCl; 1 CaCl_2_; 1 MgSO_4_; 25 HEPES; 1 KH_2_PO_4_ and 10 glucose, adjusted to pH 7.4 and previously oxygenated for 15 min with O_2_). The medium used for slices was exchanged every 15 min with fresh saline at room temperature (equilibration period) for up to 60 min. It is important to note that this is an acute model of hippocampal slices, which are viable up to 5 h after preparation^57^.

### ZIKV infection of hippocampal slices

Following the equilibration period, the medium of hippocampal slices was removed and replaced with 0.3 mL fresh saline containing different PFU of ZIKV (10^2^–10^6^ or an equivalent volume of non-infection control medium) or YFV (10^2^–10^6^ or an equivalent volume of non-infection control medium) for an adsorption period of 1 h at 37 °C. Subsequently, the inoculum was exchanged with fresh saline medium and hippocampal slices were maintained at 37 °C for 1 h or 2 h. The extracellular medium and slices were harvested at these two time points for subsequent analyses.

### Membrane integrity and metabolic activity assays

*MTT reduction assay* slices were treated with 0.5 mg/mL of MTT for 30 min at 30 °C. The MTT formazan was dissolved in DMSO^56^. Absorbance values were measured at 560 and 650 nm. Results are expressed as percentages of the non-infection control value.

*Lactate dehydrogenase assay* the release of the enzyme lactate dehydrogenase was assessed measuring its activity in the extracellular medium (100 μL) of slices using a commercial UV assay from Bioclin (Brazil). Results are expressed as percentages of the non-infection control value.

*Neuron-specific enolase (NSE) activity* extracellular NSE was measured using an eletrochemiluminescent assay purchased from Roche Diagnostics. The assay is a double sandwich that uses an antibody anti-NSE bound with ruthenium, which produces light emission when excited. The reaction and quantification were performed by the equipment Elecsys-2010 (Roche Diagnostics Corporation). Results are expressed as percentages of the non-infection control value.

### Cytokine measurement

Cytokine levels were measured in the extracellular medium using ELISA kits for TNFα (Peprotech), IL1β, IL6, IL10 and MCP1 (Invitrogen)^31^. The results are expressed in pg/mL and the average minimum sensitivity of the ELISA kits detection was: 25.0 pg/mL for TNFα; 12 pg/mL for IL1β; 16 pg/mL for IL6; 3 pg/mL for IL10; and 5 pg/mL for MCP1.

### Trophic factor release

BDNF and GDNF levels were measured in the extracellular medium, using commercial ELISA kits from Invitrogen and R&D Systems, respectively^31^. The results are expressed in pg/mL. The ELISA kits detect a minimum of 12 pg/mL for BDNF and 31.2 pg/mL for GDNF.

### S100B secretion measuremen*t*

S100B secretion was measured by an enzyme-linked immunosorbent assay, as previously described^58^. Briefly, 50 µL of extracellular medium from slices and 50 µL of Tris buffer were incubated for 2 h on a microtiter plate previously coated with monoclonal anti-S100B (SH-B1; Sigma-Aldrich). Next, the samples were incubated with polyclonal anti-S100B (Dako) for 30 min, and then, peroxidase-conjugated anti-rabbit antibody (Amersham) was added for a further 30 min incubation period. A colorimetric reaction with o-phenylenediamine (Sigma-Aldrich) was observed at 492 nm. Results are expressed as percentages of the non-infection control value.

### GSH levels

GSH levels were fluorometrically assessed as previously described^58^. Hippocampal slices were suspended in a 100 mM sodium phosphate buffer with 140 mM KCl (pH 8.0) containing 5 mM EDTA. After that, protein was precipitated with 1.7% metaphosphoric acid. The supernatant was assayed with o-phthaldialdehyde (at a concentration of 1 mg/mL methanol; Sigma-Aldrich) at room temperature for 15 min. Fluorescence was measured using excitation and emission wavelengths of 350 and 420 nm, respectively. A calibration curve was performed with standard GSH (Sigma-Aldrich) solutions at concentrations ranging from 0 to 500 μM. Results are expressed as percentages of the non-infection control value.

### RNA extraction and quantitative RT-PCR

Total RNA was isolated from hippocampal slices (control and infected) using TRIzol Reagent (Invitrogen, Carlsbad, CA). Extracted RNA (1 μg) was submitted to cDNA synthesis by High Capacity cDNA Reverse Transcription Kit (Applied Biosystems, Thermo Fisher Scientific). Quantitative PCR for viral copies determination was performed in total volumes of 12.5 µL containing 3 µL of cDNA, 6.25 µL of 2× Platinum quantitative PCR SuperMix-UDG (Invitrogen—Life Technologies), 200 nM each of forward and reverse primers (ZIKV 1086 and ZIKV 1162c^59^; RP-YFV and FP-YFV^60^). Amplification was carried out in a StepOne Real-Time PCR system (Applied Biosystems, Thermo Fisher Scientific) under the following conditions; uracil DNA glycosylase (UDG) incubation at 50 °C for 2 min; initial denaturation and Platinum Taq activation at 95 °C for 2 min, followed by 40 cycles of amplification (15 s at 95 °C and 30 s at 60 °C). All real-time assays were performed in triplicate and results depict the means of these triplicate values. Viral RNA quantification was estimated in relation to ZIKV or YFV standard curves (10^8^ to 10 copies) and data analysis was performed with the StepOne software v2.2.2.

For neural gene expression, the messenger RNAs (mRNAs) encoding TNFα (#Rn99999017_m1), TNF receptor 1 (TNFR1; #Rn01492348_m1), IL1β (#Rn00580432_m1), IL1 receptor type I (IL1R1; #Rn00565482_m1), HMGB1 (#Rn02377062_g1), COX2 (#Rn01483828_m1), TLR2 (#Rn02133647_s1), TLR4 (#Rn00569848_m1), NFκB p65 (#Rn01502266_m1), NFκB p50 (#Rn01399572_m1), Nrf2 (#Rn00582415_m1), HO1 (#Rn01536933_m1), iNOS (#Rn00561646_m1), SOD1 (#Rn00566938_m1), SOD2 (#Rn00690588_g1), GCL (#Rn00689046_m1), PI3K (#Rn01769524_m1), Akt (#Rn00442194_m1), p21 (#Rn 00589996_m1), SIRT1 (#Rn01428096_m1), VEGF (#Rn01511602_m1), adenosine receptors A1 (#Rn00567668_m1), A2a (#Rn00583935_m1), A2b (#Rn00567697_m1), A3 (#Rn00563680_m1), GFAP (#Rn00566603_m1), vimentin (#Rn00667825_m1), nestin (#Rn00564394_m1), AQP4 (#Rn00563196_m1), GLT1 (#Rn00691548_m1), GLAST (#Rn00570130_m1), GS (#Rn01483107_m1), EAAC1 (#Rn00564705_m1), and β-actin (#Rn00667869_m1) were quantified using the TaqMan real-time RT-PCR system using inventory primers and probes purchased from Applied Biosystems (Thermo Fisher Scientific), as referred for each gene^31^. Target mRNA levels were normalized to β-actin levels. Results were analyzed employing the 2^−ΔΔCt^ method and expressed relative to the levels of non-infection control conditions (1 h).

### Western blotting analysis

Hippocampal slices were lysed in a solution containing 4% SDS, 2 mM EDTA, 1 mM Na_3_VO_4_, 10 mM NaF and 50 mM Tris–HCl (pH 6.8). Samples were separated by SDS/PAGE (20 μg protein per sample) and transferred to nitrocellulose membranes using a semi dry blotting apparatus (1.2 mA/cm^2^; 1 h). The membranes were blocked with 2% albumin in Tris-buffered saline with Tween 20 (T-TBS) and then incubated overnight (4 °C) with anti-PI3K (1:2000, Cell Signaling), anti-Akt (1:2000; Cell Signaling), anti-NMDAR1 (1:5000; Millipore), anti-GFAP (1:5000; Sigma-Aldrich), anti-synaptophysin (1:5000; Millipore), anti-tubulin βIII (1:5000; Abcam), or anti-actin (1:5000; Millipore). β-actin was used as a loading control. Subsequently, the membranes were incubated for 1 h at room temperature with horseradish peroxidase-conjugated anti-mouse IgG or anti-rabbit IgG (1:10,000; GE Healthcare) for 1 h. The chemiluminescence signal was detected in an Image Quant LAS4010 system (GE Healthcare) using an ECL kit (GE Healthcare). Results are expressed as percentages relative to non-infection control conditions.

### Statistical analyses

Differences among groups were statistically analyzed using one-way analysis of variance (ANOVA), followed by Tukey’s test. All analyses were performed using the GraphPad Prism 7 (GraphPad Software, Inc., La Jolla, CA, USA). Values of *P* < 0.05 were considered significant. Heatmaps were created using GraphPad Prism 7 and Heatmapper^61^.

## Supplementary Information

Supplementary Information.

## Data Availability

The datasets generated during and/or analyzed during the current study are available from the corresponding author on reasonable request.
