# Supplementary material for: Zika virus exposure affects neuron-glia communication in the hippocampal slices of adult rats
Source: Sci Rep. 2020 Dec 10;10:21604. doi: 10.1038/s41598-020-78735-y (PMC7729948; doi:10.1038/s41598-020-78735-y)
Supplement: Supplementary file 1 — Supplementary Information. [file 41598_2020_78735_MOESM1_ESM.pdf]

Supplementary Information

Manuscript title: Zika virus exposure affects neuron-glia communication in the hippocampus of adult rats

Author list: Larissa Daniele Bobermin[*] [1], André Quincozes-Santos[*] [1,2], Camila Leite Santos[1], Ana Paula M. Varela[3], Thais F. Teixeira[3], Krista Minéia Wartchow[1], Lílian Juliana Lissner[1], Amanda da Silva[1], Natalie K. Thomaz[1], Lucélia Santi[4,5,6], Walter O. Beys-da-Silva[4,5,6], Paulo M. Roehe[3], Patrícia Sesterheim[7], Jorge A. Guimarães[5,6], Carlos-Alberto Gonçalves[1,2], Diogo Onofre Souza[1,2]

[*] These authors contributed equally to this work.

[1]Programa de Pós-Graduação em Ciências Biológicas: Bioquímica, Instituto de Ciências Básicas da Saúde, Universidade Federal do Rio Grande do Sul, Porto Alegre, RS, Brazil

[2]Departamento de Bioquímica, Instituto de Ciências Básicas da Saúde, Universidade Federal do Rio Grande do Sul, Porto Alegre, RS, Brazil

[3]Departamento de Microbiologia, Imunologia e Parasitologia, Instituto de Ciências Básicas da Saúde, Universidade Federal do Rio Grande do Sul, Porto Alegre, RS, Brazil

[4]Faculdade de Farmácia, Universidade Federal do Rio Grande do Sul, Porto Alegre, RS, Brazil

[5]Centro de Pesquisa Experimental, Hospital de Clínicas de Porto Alegre, Porto Alegre, RS, Brazil

[6]Programa de Pós-Graduação em Biologia Celular e Molecular, Centro de Biotecnologia, Universidade Federal do Rio Grande do Sul, Porto Alegre, RS, Brazil

[7]Centro de Cardiologia Experimental, Instituto de Cardiologia/Fundação Universitária de Cardiologia, Porto Alegre, RS, Brazil

André Quincozes-Santos

Departamento de Bioquímica

Programa de Pós-Graduação em Ciências Biológicas: Bioquímica

Instituto de Ciências Básicas da Saúde

Universidade Federal do Rio Grande do Sul

Rua Ramiro Barcelos, 2600 – Anexo

Bairro Santa Cecília

90035–003 Porto Alegre, RS, Brazil

Phone: +55 51 3308 5567

E-mail: andrequincozes@ufrgs.br

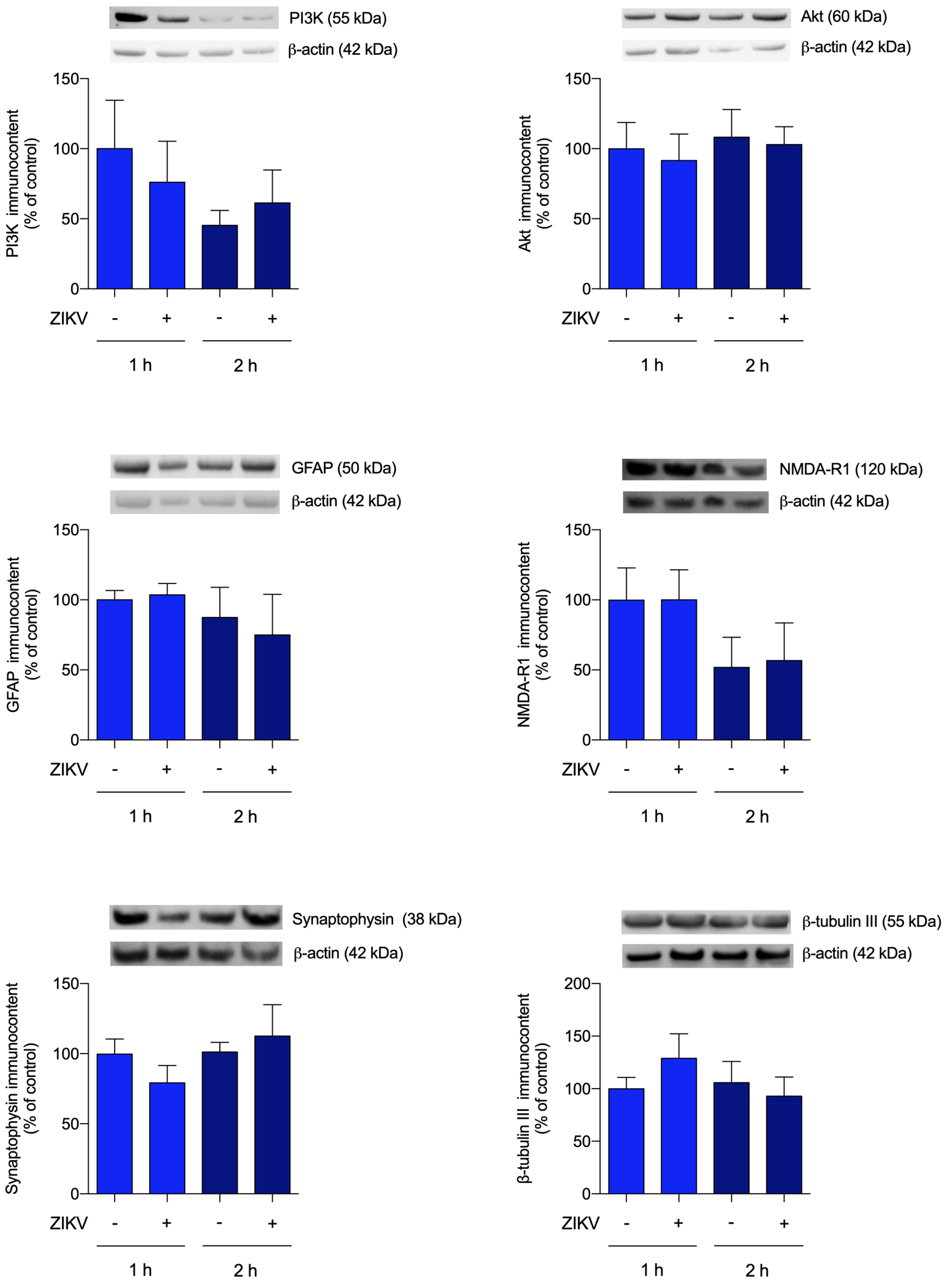

Figure S1. Effects of ZIKV on immunocontent of proteins related to neuronal and glial functions. Hippocampal slices were infected with 10^5 PFU of ZIKV (non-infection controls were simultaneously carried out). Western blotting analysis for (a) PI3K; (b) Akt; (c) GFAP; (d) NMDA-R1; (e) synaptophysin; (f) β-tubulin III were performed in the hippocampal slices after 1 h or 2 h incubation. Bars represent means ± SEM of at least three experimental determinations, analyzed by one-way ANOVA followed by Tukey's test. Representative western blotting images are shown in the graphs.

PI3K (55 kDa)

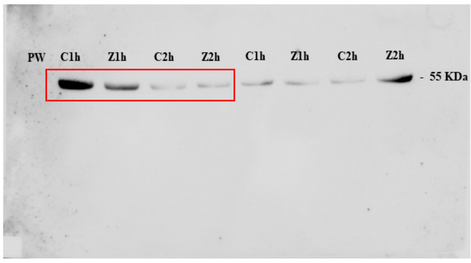

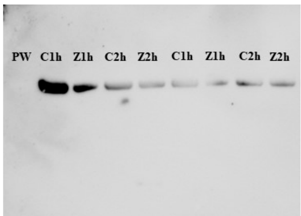

PW, Protein weight

C1h, Control 1 h

Z1h, ZIKV 1 h

C2h, Control 2 h

Z2h, ZIKV 2 h

β-actin (42 kDa) for PI3K analysis

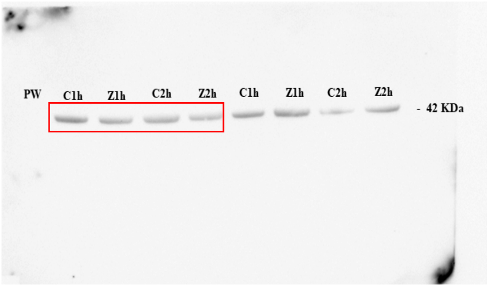

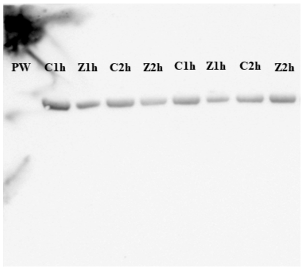

Figure S2. Full-length western blot membranes for PI3K detection and the respective β-actin controls. Red boxes highlight the bands used as representative images in the Figure S1.

Akt (60 kDa)

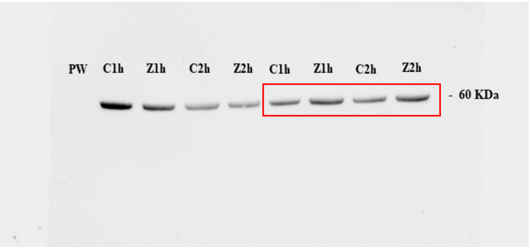

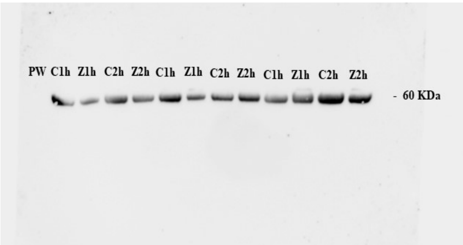

PW, Protein weight

C1h, Control 1 h

Z1h, ZIKV 1 h

C2h, Control 2 h

Z2h, ZIKV 2 h

β-actin (42 kDa) for Akt analysis

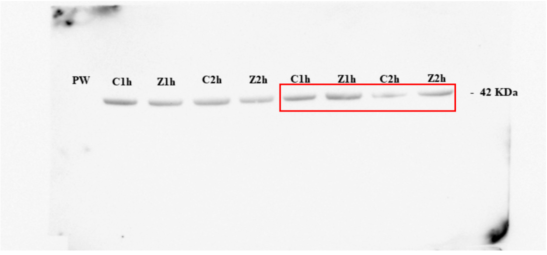

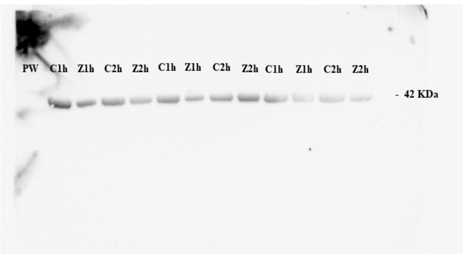

Figure S3. Full-length western blot membranes for Akt detection and the respective β-actin controls. Red boxes highlight the bands used as representative images in the Figure S1.

GFAP (50 kDa)

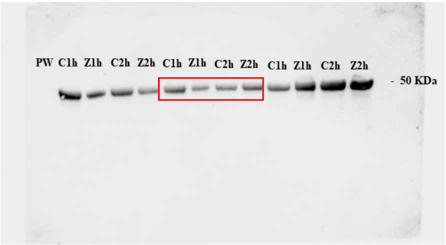

PW, Protein weight

C1h, Control 1 h

Z1h, ZIKV 1 h

C2h, Control 2 h

Z2h, ZIKV 2 h

β-actin (42 kDa) for GFAP analysis

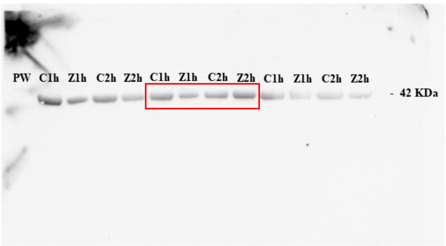

Figure S4. Full-length western blot membranes for GFAP detection and the respective β-actin controls. Red boxes highlight the bands used as representative images in the Figure S1.

NMDA-R1 (60 kDa)

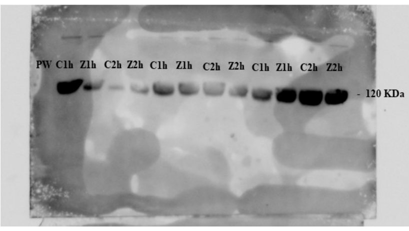

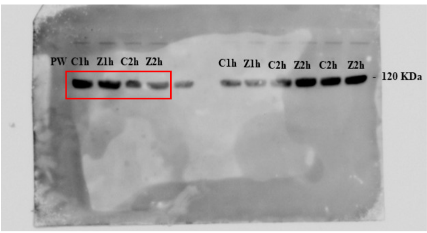

PW, Protein weight

C1h, Control 1 h

Z1h, ZIKV 1 h

C2h, Control 2 h

Z2h, ZIKV 2 h

β-actin (42 kDa) for NMDA-R1 analysis

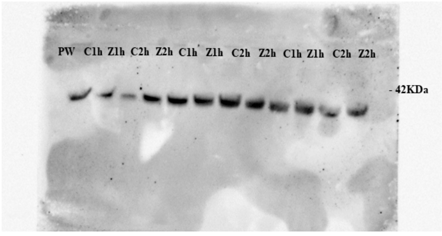

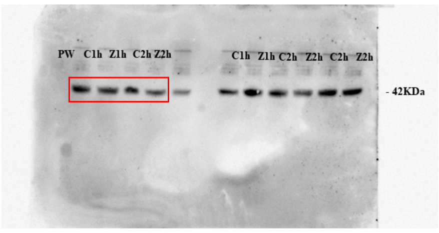

Figure S5. Full-length western blot membranes for NMDA-R1 detection and the respective β-actin controls. Red boxes highlight the bands used as representative images in the Figure S1.

Synaptophysin (38 kDa)

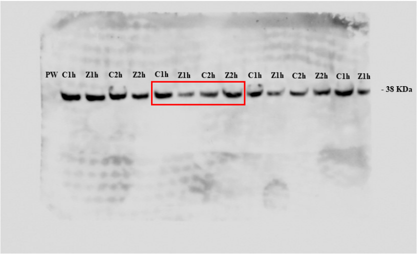

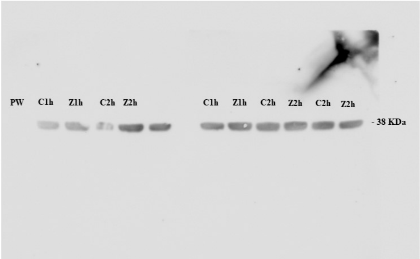

PW, Protein weight

C1h, Control 1 h

Z1h, ZIKV 1 h

C2h, Control 2 h

Z2h, ZIKV 2 h

β-actin (42 kDa) for synaptophysin analysis

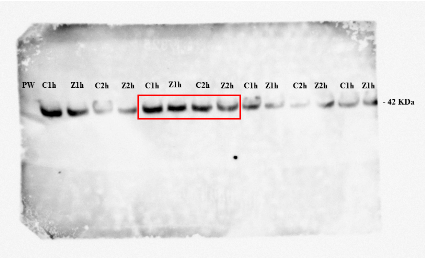

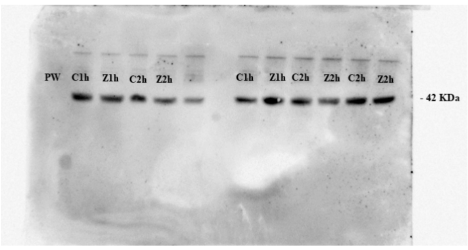

Figure S6. Full-length western blot membranes for synaptophysin detection and the respective β-actin controls. Red boxes highlight the bands used as representative images in the Figure S1.

β-tubulin III (55 kDa)

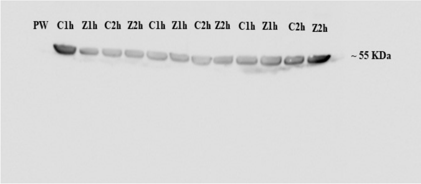

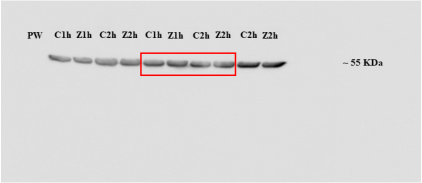

PW, Protein weight

C1h, Control 1 h

Z1h, ZIKV 1 h

C2h, Control 2 h

Z2h, ZIKV 2 h

β-actin (42 kDa) for β-tubulin III analysis

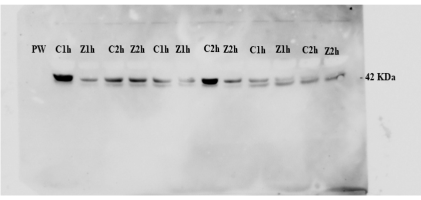

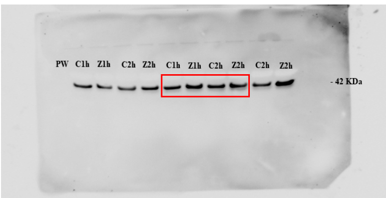

Figure S7. Full-length western blot membranes for β-tubulin III detection and the respective β-actin controls. Red boxes highlight the bands used as representative images in the Figure S1.
